# Effects of Cryotherapy and Thermotherapy Using an E-TEET on Pain, Stress, and Satisfaction Among Patients and Healthcare Providers During Intravenous Catheterization: A Randomized Controlled Trial

**DOI:** 10.3390/nursrep16010017

**Published:** 2026-01-07

**Authors:** Bosong Kim, Soukyoung Kim, Jihoo Her, Yu Jin Lee, Myung-Haeng Hur

**Affiliations:** 1 Daejeon Eulji University Hospital, Daejeon 35233, Republic of Korea; bsong22@eulji.ac.kr; 2 College of Nursing, Eulji University 712, Dongil-ro, Uijeongbu-si 11759, Republic of Korea; ksy@eulji.ac.kr (S.K.); icejin224@naver.com (Y.J.L.); 3 Department of Nursing, Gimcheon University, Gimcheon 39528, Republic of Korea; jihoo@gimcheon.ac.kr

**Keywords:** cryotherapy, thermotherapy, intravenous catheterization, pain, stress, tourniquets, nursing

## Abstract

**Background**: Intravenous catheterization is a common nursing procedure, although it is invasive and may cause pain and stress. Non-pharmacological interventions such as cryotherapy and thermotherapy have been explored, but practical and effective options remain limited. **Purpose**: This study aimed to evaluate the effects of cryotherapy and thermotherapy using the Enhanced Thermoelectric Element Tourniquet (E-TEET) a device equipped with a temperature-controlled plate and wireless charging on pain, stress, and patient satisfaction during intravenous catheterization. **Methods**: A randomized controlled trial was conducted involving 128 adult inpatients scheduled for preoperative intravenous catheterization. Participants were randomly assigned to one of four groups: cryotherapy (n = 31), thermotherapy (n = 31), control (E-TEET without temperature, n = 33), or comparison (latex tourniquet, n = 33). Pain and stress levels were measured using- the Numeric Rating Scale (NRS), along with pulse rate and oxygen saturation. Post-procedure satisfaction was also evaluated. **Results**: No significant differences were observed among the groups in terms of pain, pulse rate, or oxygen saturation. However, the cryotherapy group exhibited significantly lower stress levels and higher satisfaction compared to the comparison group (*p* < 0.05). Furthermore, Healthcare provider Satisfaction was significantly higher in the cryotherapy group than in the control group (*p* < 0.05). **Conclusions**: Cryotherapy using the E-TEE Tourniquet effectively reduced stress and improved satisfaction during intravenous catheterization, supporting its use as a feasible non-pharmacological intervention. Further studies are needed to standardize intervention parameters and validate findings across populations.

## 1. Introduction

Pain is defined as an unpleasant sensory and emotional experience associated with actual or potential tissue damage and is often accompanied by psychological stress [1]. It arises from a complex interaction between physiological sensations and subjective emotional factors following noxious stimuli [1,2].

In clinical settings, particularly during invasive procedures, pain is typically acute in nature and may be accompanied by heightened psychological and physiological stress responses. Hospitalized patients frequently undergo repeated venipuncture for essential medical procedures such as blood sampling, fluid and nutrient administration, and medication delivery, which can induce pain and stress. Repeated exposure to such procedures may heighten psychological distress and contribute to negative perceptions of the hospital environment [3,4]. Such experiences may increase stress levels and negatively influence satisfaction with care among both patients and healthcare providers. Patient satisfaction during venipuncture reflects not only pain intensity but also psychological comfort and perceived quality of care [5]. In addition to pain and stress, satisfaction with intravenous catheterization reflects patients’ overall care experience and practitioners’ procedural convenience and is therefore considered an important outcome in evaluating nursing interventions [5]. Interventions that reduce stress may thus contribute to greater satisfaction, even when pain reduction is limited. Therefore, there is a growing need for effective nursing interventions that can alleviate pain caused by invasive procedures, such as intravenous catheterization, and facilitate the nursing process [6]. Numerous studies have been conducted to identify non-pharmacological nursing interventions effective in reducing pain associated with injections and venipuncture. Previous research on pain reduction related to venipuncture has primarily focused on vulnerable populations such as children [7,8]. and hemodialysis patients requiring arteriovenous fistula puncture [9,10,11,12]. Various interventions have been explored, including vapocoolant sprays [13,14,15], lidocaine patches or sprays [16,17], EMLA cream [18], distraction techniques [7,19], and aromatherapy [20].

Cryotherapy and thermotherapy are among the most common non-pharmacological nursing interventions for pain relief and are widely used in clinical settings [21]. Cryotherapy alleviates pain and inflammation through vasoconstriction, reduced metabolic rate, and inhibition of nerve conduction, while also promoting muscle relaxation and edema reduction [22]. Studies have demonstrated that the application of ice packs can significantly decrease subjective pain perception [23]. Conversely, thermotherapy inhibits pain signal transmission based on the gate control theory by promoting nerve conduction and vasodilation, which relaxes muscles and relieves stiffness caused by ischemia. Research applying thermotherapy has shown its effectiveness in reducing pain during arteriovenous fistula puncture in hemodialysis patients [24] and intravenous injections in pediatric emergency patients [25]. In addition to pain relief, these interventions may also reduce procedure-related stress and improve satisfaction with the care experience for both patients and healthcare providers. However, both cryotherapy and thermotherapy are not routinely implemented in clinical practice due to the inconvenience of application methods and insufficient evidence of their effectiveness. Therefore, it is necessary to simplify and improve their application for broader clinical use.

To address these challenges, a Thermoelectric Element (TEE) has recently been developed as a more efficient method for delivering controlled heat and cold stimuli [6]. The TEE directly converts electrical energy into thermal energy through thermoelectric conversion and is capable of simultaneously providing both heating and cooling [6].

The Thermoelectric Element Band (TEE Band), designed for easy application to the wrist, enables the delivery of cryotherapy or thermotherapy while a tourniquet is applied to the upper arm during venipuncture, and previous studies reported that it was effective in reducing venipuncture-related pain and improving patient satisfaction [6]. Subsequently, studies using the Thermoelectric Element Tourniquet (TEE Tourniquet), which integrates temperature control and tourniquet functions into a single apparatus, demonstrated that cryotherapy and thermotherapy significantly reduced pain and were positively received by both patients and healthcare providers [24,26].

However, previous studies reported inconsistent findings regarding the relative effectiveness of thermotherapy and cryotherapy, with some demonstrating the efficacy of thermotherapy [26] and others reporting greater effects of cryotherapy [24]. These conflicting results indicate the need for further investigation to clarify the distinct effects of cold and heat stimulation. In addition, the previous M-TEET had practical limitations, including the need for optimized plate size for efficient temperature transfer and the inconvenience and aesthetic drawbacks of a wired power supply.

To overcome these issues, the present study utilized the Enhanced Thermoelectric Element Tourniquet (E-TEET), an improved device designed with an adjustable plate area to maintain tourniquet function while providing effective cryotherapy and thermotherapy. The E-TEET also incorporates wireless charging to enhance usability and aesthetics. By integrating cryotherapy, thermotherapy, and tourniquet functionality into a single device, the E-TEET aims to address the limitations of previous TEE-based interventions and to improve usability, effectiveness, and practical applicability during intravenous catheterization.

Given these limitations and inconsistent findings in previous studies, further investigation is warranted to clarify the effects of cryotherapy and thermotherapy using an improved thermoelectric device in clinical practice.

The study aimed to examine the effects of applying the E-TEET, which incorporates an enhanced thermoelectric element, on pain, stress, and patient satisfaction during intravenous catheterization, with healthcare provider Satisfaction assessed as a supplementary outcome.

Based on this aim, the following hypotheses were tested:

**Hypothesis 1 (Pain). **  *There will be a significant difference in perceived pain during intravenous catheterization among the four groups: cryotherapy using the E-TEET, thermotherapy using the E-TEET, E-TEET alone, and a latex tourniquet.*

**Hypothesis 2 (Stress).** 
*There will be a significant difference in patient stress during intravenous catheterization among the four groups: cryotherapy using the E-TEET, thermotherapy using the E-TEET, E-TEET alone, and a latex tourniquet.*

**Hypothesis 3 (Patient Satisfaction).** 
*There will be a significant difference in patient satisfaction with intravenous catheterization among the four groups: cryotherapy using the E-TEET, thermotherapy using the E-TEET, E-TEET alone, and a latex tourniquet.*

**Hypothesis 4 (Healthcare Provider Satisfaction).** 
*There will be a significant difference in healthcare provider satisfaction during intravenous catheterization among the four groups: cryotherapy using the E-TEET, thermotherapy using the E-TEET, E-TEET alone, and a latex tourniquet.*

By testing these hypotheses, this study aimed to contribute evidence supporting the potential clinical use of the E-TEET as a non-pharmacological nursing intervention during intravenous catheterization.

## 2. Materials and Methods

### 2.1. Study Design

**Figure 1:**
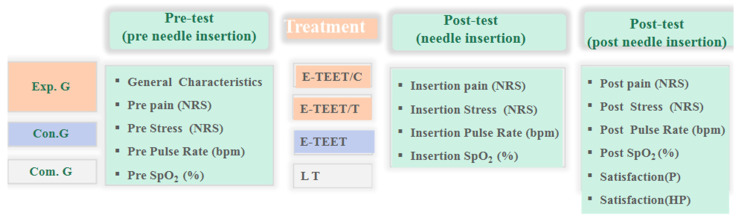
Study design.

This study was a randomized controlled trial designed to evaluate the effects of cryotherapy (E-TEET/C) and thermotherapy (E-TEET/T) using the Enhanced Thermoelectric Element Tourniquet (E-TEET; Korea) on pain, stress, and satisfaction during intravenous catheterization among hospitalized patients requiring an 18-gauge angio-catheter. Participants were randomly assigned to one of four groups: cryotherapy, thermotherapy, control (E-TEET without temperature application), or comparison (latex tourniquet, LT). The study design is illustrated in Figure 1.

E-TEET/C: Enhanced Thermoelectric element Tourniquet–Cryotherapy group. E-TEET/T: Enhanced Thermoelectric element Tourniquet–Thermotherapy group. Con. G: Control Group (only E-TEE Tourniquet). Com. G: Comparison Group (Latex Tourniquet, LT). NRS: Numeral rating scale. bpm: beats per min. SpO2: Saturation of Percutaneous Oxygen. Pre pain: Pre-needle pain after tourniquet. Insertion pain: Needle insertion pain. Post pain: Post-needle insertion pain. Pre pulse rate: Pre-needle pulse rate after tourniquet. Insertion pulse rate: Needle insertion pulse rate. Post pulse rate: Post-needle insertion pulse rate. Pre SpO2: Pre-needle SpO2 after tourniquet. Insertion SpO2: Needle insertion SpO2. Post SpO2: Post-needle insertion SpO2. Pre Stress: Pre-needle stress after tourniquet. Insertion Stress: Needle insertion stress. Post Stress: Post-needle insertion stress. Satisfaction (P): Patient satisfaction. satisfaction. (HP): Healthcare provider satisfaction.

### 2.2. Participants

Participants were adult inpatients at E University Hospital located in D City who required intravenous catheterization for preoperative purposes.

Inclusion criteria were as follows: (1) inpatients aged 20–85 years who required insertion and maintenance of an 18-gauge angio-catheter; (2) intact skin at the catheterization site; (3) a history of intravenous catheterization within the previous six months; and (4) voluntary consent to participate.

Exclusion criteria included the following: (1) use of medications that could affect pain or stress responses; (2) the presence of severe bleeding or infection; and (3) inability to provide informed consent due to serious illness or cognitive impairment.

### 2.3. Sample Size Calculation and Random Allocation

The sample size was determined based on a previous study on pain management interventions during venipuncture [24,26]. Using G*Power 3.1.9.7 [27], an F-test (ANOVA) with an effect size of 0.3, significance level (α) of 0.05, power of 0.80 [28], and four groups yielded a required sample size of 128. Considering a potential 10% dropout rate, 140 participants were recruited and randomly assigned to one of four groups by block randomization (block size = 4, 1:1:1:1 allocation ratio): cryotherapy (n = 35), thermotherapy (n = 35), control (n = 35), or comparison (n = 35).

**Figure 2:**
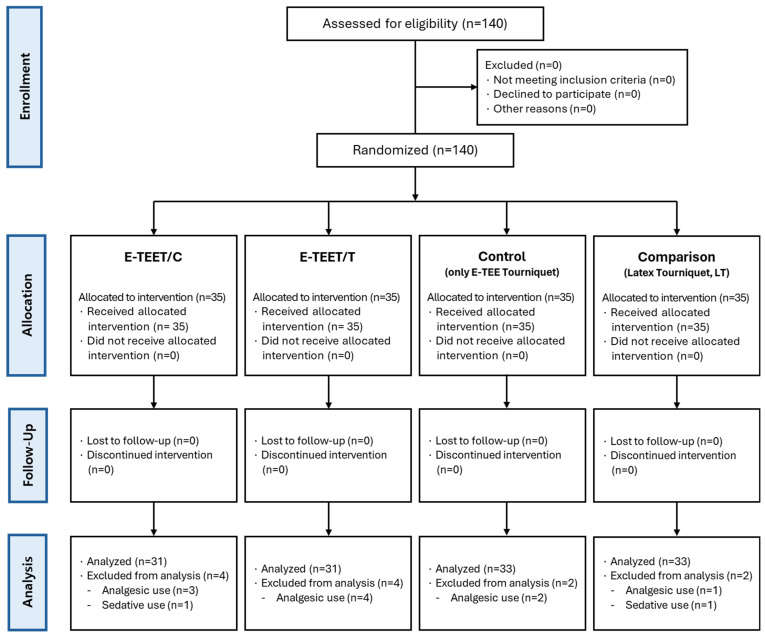
Flow diagram.

The block randomization sequence (block size = 4, fixed) was pre-generated in Excel before participant enrollment to ensure allocation concealment. All eligible participants agreed to participate, likely due to the noninvasive nature of the intervention. Twelve participants were excluded from the final analysis due to medication use that could influence pain or stress responses: cryotherapy (n = 4), thermotherapy (n = 4), control (n = 2), and comparison (n = 2). Thus, the final analysis included 128 participants: cryotherapy (n = 31), thermotherapy (n = 31), control (n = 33), and comparison (n = 33) (Figure 2).

To prevent allocation bias, block randomization (block size = 4) with opaque sealed envelopes was used, and group assignment was concealed from both participants and researchers. The research assistant, who performed the intravenous insertion, was blinded to both the temperature condition and the group assignment. The Excel allocation file was password-protected and securely managed on a research-designated computer.

E-TEET/C Group: Enhanced Thermoelectric element Tourniquet–Cryotherapy group. E-TEET/T Group: Enhanced Thermoelectric element Tourniquet–Thermotherapy group. Con G: Control Group (only E-TEE Tourniquet). Com G: Comparison Group (Latex Tourniquet, LT).

### 2.4. Preparation of Research Assistants

To ensure consistency in data collection, a research assistant was trained by the principal investigator regarding the study’s purpose, procedures, and intervention protocols. The research assistant—one registered nurse with 14 years of clinical experience—performed all intravenous catheterizations to control for skill-related variability.

### 2.5. Instruments

(1) Pain
  Pain was assessed using both subjective (self-reported) and objective (physiological) measures.
  (1) *Perceived pain:* Perceived pain was measured using the Numeric Rating Scale (NRS), a horizontal 11-point scale ranging from 0 (“no pain”) to 10 (“worst possible pain”). Participants indicated their pain intensity by marking the scale, with higher scores representing greater pain intensity.
  (2) *Physiological indicators of pain (pulse rate and oxygen saturation):* Objective indicators of pain were measured using a pulse oximeter (Pulse Oximeter 32MX, Nellcor, Covidien, Boulder, CO, USA) to record pulse rate (bpm) and oxygen saturation (%).

(2) Stress

Perceived stress was measured using the Numeric Rating Scale (NRS), ranging from 0 (“no stress at all”) to 10 (“extreme stress”). Participants marked their perceived stress level on the scale, with higher scores indicating greater stress intensity.

(3) Satisfaction with the E-TEET
  (1) *Patient satisfaction:* Participant satisfaction with the E-TEET was assessed using a modified questionnaire developed in a previous study [6]. The instrument consisted of nine items rated on a 5-point Likert scale. Reliability of the instrument was Cronbach’s α = 0.87 in the previous study and 0.95 in the present study.
  (2) *Healthcare Provider satisfaction:* Healthcare provider Satisfaction was also measured using a modified version of a previously validated questionnaire [6], consisting of eight items on a 5-point Likert scale. Reliability was Cronbach’s α = 0.86 in the prior study and 0.87 in the present study.
(4) Baseline characteristics

Perceived vein status, pain tolerance, previous pain during intravenous insertion (NRS), and previous stress (NRS) were assessed as baseline variables using single-item self-reported questions.

Perceived vein status reflected participants’ subjective perception of their venous condition. Pain tolerance represented individuals’ self-reported general response to pain in daily life. Previous pain and stress were evaluated based on participants’ prior experiences with intravenous insertion.

These variables were collected solely to assess baseline homogeneity among groups and were not used as primary outcome measures.

### 2.6. Experimental Procedures

The E-TEE Tourniquet used in this study was designed with a 5.5 × 3.5 cm thermoelectric plate, a wireless, rechargeable power system, and an aesthetically improved elastic band. Based on previous studies examining thermoelectric element–based cryotherapy and thermotherapy during venipuncture [6,24,26], the device was applied approximately 10–12 cm above the insertion site and activated in Cold or Hot mode for 10–30 s during intravenous catheterization. All intravenous catheterizations were performed by a trained research assistant, a registered nurse with 14 years of clinical experience. Adverse events were monitored by direct observation during the procedure and by asking participants to report any unexpected symptoms immediately after intravenous catheterization.

(1) Temperature and Duration Settings

The E-TEE Tourniquet provided adjustable cold and hot modes. Based on previous studies [5], safe therapeutic ranges were set at 0–10 °C for cryotherapy and 40–45 °C for thermotherapy. Prior to venipuncture, participants were observed for 5 s to assess temperature tolerance, and intensity was reduced if discomfort occurred.

The tourniquet was applied approximately 10–12 cm above the puncture site, and intravenous catheterization was performed while maintaining the assigned thermal condition. Temperature was applied for 10–30 s, depending on catheterization success. Temperature intensity and application duration were individually adjusted to ensure participant safety and to reflect routine clinical practice; this variability was considered in the interpretation of the study findings.

(2) Application Procedure

Participants who voluntarily enrolled following hospital recruitment notices were briefed on the study by a research assistant and provided written informed consent prior to participation. After consent was obtained, the research assistant administered baseline questionnaires on general characteristics and previous experiences with intravenous catheterization.

The research assistant then positioned the participant comfortably, applied the assigned tourniquet, and attached a pulse oximeter to the contralateral hand. Baseline pulse rate, oxygen saturation, pre-procedure pain, and pre-procedure stress were recorded. According to group allocation, the research assistant adjusted the E-TEE Tourniquet temperature settings and applied the assigned thermal condition after a 5 s tolerance check.

Intravenous catheterization was performed by the researcher using an 18-gauge angio-catheter, while the assigned thermal condition was maintained. The tourniquet was removed immediately after successful insertion. Group-specific interventions were as follows:

(1) Cryotherapy group (E-TEET/C): Cold mode (0–10 °C) applied for 10–30 s;
(2) Thermotherapy group (E-TEET/T): Hot mode (40–45 °C) applied for 10–30 s;
(3) Control group: E-TEE Tourniquet applied without temperature activation;
(4) Comparison group: Conventional latex tourniquet applied without thermal control.

Pulse rate and oxygen saturation were continuously monitored during venipuncture and recorded again after catheterization. Following the procedure, participants completed questionnaires assessing insertion pain, post-procedure pain, stress, and satisfaction with the tourniquet. Healthcare provider satisfaction with the E-TEE Tourniquet was also assessed by the research assistant (Supplementary Material S2).

### 2.7. Data Collection

Data were collected at E University Hospital in D City from 12 February to 11 April 2025. Prior to data collection, the researchers completed human subject research ethics training and obtained approval from the Institutional Review Board (IRB) of E University Hospital (Approval No. EMC IRB 2024-08-016-003). The study was also registered with the Clinical Research Information Service (CRIS) (Registration No. KCT0010186). Written permission was obtained from the hospital’s nursing department, and recruitment notices were posted on ward bulletin boards.

### 2.8. Data Analysis

Data were analyzed using SPSS WIN 30.0 software. Descriptive statistics (frequency, mean, and percentage) were used to summarize participant characteristics. Homogeneity tests among the four groups were conducted using the χ^2^ test and ANOVA. Differences in pain, stress, and satisfaction among the groups were examined using ANCOVA, with age included as a covariate due to non-homogeneity across groups. When significant differences were identified, Bonferroni post hoc analyses were performed. The reliability of the satisfaction instruments was assessed using Cronbach’s alpha coefficients.

### 2.9. Ethical Considerations

The researchers completed certified training in human subject research ethics through the National Bioethics Policy Institute. The study protocol was approved by the Institutional Review Board of E University Hospital (EMC IRB 2024-08-016-003) and registered with the Clinical Research Information Service (CRIS; KCT0010186). This study was conducted in accordance with the ethical principles of the Declaration of Helsinki. As a non-pharmacological nursing intervention study, formal Good Clinical Practice (GCP) certification was not required.

Prior to participation, all subjects were informed of the study purpose, procedures, potential risks, and compensation, and written informed consent was obtained. Participants were informed of their right to withdraw at any time without penalty, and refusal or withdrawal did not affect their treatment or nursing care. All collected data were anonymized and stored securely, and personal information was used solely for research purposes in accordance with institutional regulations.

To minimize undue influence, study information and informed consent were provided by trained research staff not involved in routine clinical care or intravenous catheter insertion. Participants were involved only as study subjects and were not involved in the design, conduct, analysis, or reporting of the research.

### 2.10. Study Governance

The trial was overseen by the principal investigator, who was responsible for study design, protocol adherence, and data integrity. The institutional review board approved the study protocol and monitored ethical compliance throughout the study period. Given the single-center, low-risk nature of the intervention, no separate trial steering committee was established.

## 3. Results

### 3.1. Homogeneity Test for the Participant’s General Characteristics and Previous Dependent Variables

A total of 128 participants were included in the analysis (cryotherapy, n = 31; thermotherapy, n = 31; control, n = 33; comparison, n = 33). The results of the homogeneity tests for general characteristics and baseline variables are presented in Table 1. No significant differences were found among the four groups in gender, height, weight, venipuncture experience within the last six months, perceived vascular condition, pain tolerance, or prior venipuncture-related pain and stress. However, age differed significantly among groups (*p* < 0.001); therefore, age was treated as a covariate in subsequent analyses using ANCOVA (Table 1).

### 3.2. Effects of the E-TEE Tourniquet on Pain, Stress, and Satisfaction During Intravenous Catheterization

#### 3.2.1. Pain

The results for perceived pain according to tourniquet type are presented in Table 2. Because age differed significantly among groups (F = 8.098, *p* < 0.001), it was controlled as a covariate.

The baseline pain score (pre-test pain) was 0.42 ± 0.77 in the cryotherapy group, 0.71 ± 0.86 in the thermotherapy group, 0.55 ± 0.75 in the control group, and 1.97 ± 1.13 in the comparison group. After adjusting for age (covariate value: 47.70 years), a significant difference was found among the four groups (F = 21.148, *p* < 0.001). However, there were no significant differences among groups in pain perceived during needle insertion (insertion pain)—4.81 ± 2.84, 4.87 ± 2.30, 5.48 ± 1.92, and 5.58 ± 1.87, respectively—or after venipuncture (post-test pain)—1.68 ± 1.25, 2.58 ± 1.79, 2.42 ± 1.33, and 3.15 ± 1.25, respectively.

The pulse rate measured before, during, and after venipuncture showed no significant group differences, with mean values ranging between 75 and 79 bpm across all groups. Likewise, oxygen saturation (SpO_2_) before, during, and after the procedure remained stable (97–98%) and did not differ significantly among groups (Table 2).

#### 3.2.2. Stress

Perceived stress scores before venipuncture (pre-test stress) were 2.90 ± 2.65, 3.13 ± 2.78, 3.73 ± 2.36, and 4.18 ± 2.43 in the cryotherapy, thermotherapy, control, and comparison groups, respectively, with no significant differences.

However, at the moment of needle insertion, stress levels differed significantly among groups (F = 3.757, *p* = 0.013): 3.68 ± 2.57 in the cryotherapy group, 4.68 ± 2.63 in the thermotherapy group, 5.85 ± 2.45 in the control group, and 5.48 ± 1.89 in the comparison group. After venipuncture, stress scores (post-test stress) were 1.23 ± 1.12, 2.06 ± 2.03, 1.55 ± 1.44, and 2.73 ± 1.53, respectively, also showing a significant difference (F = 5.967, *p* < 0.001) (Table 2).

#### 3.2.3. Patient Satisfaction

The total patient satisfaction score during intravenous catheterization differed significantly among the four groups (F = 22.235, *p* < 0.001): 35.90 ± 7.08 in the cryotherapy group, 31.68 ± 5.63 in the thermotherapy group, 28.03 ± 5.74 in the control group, and 23.21 ± 4.74 in the comparison group. Bonferroni post hoc analysis revealed that satisfaction in the cryotherapy group was significantly higher than that in the comparison group (*p* < 0.05) (Table 2).

#### 3.2.4. Healthcare Provider Satisfaction

Healthcare provider Satisfaction with the different types of tourniquets is shown in Table 2. The mean satisfaction scores were 29.52 ± 5.97 for the cryotherapy group, 26.39 ± 5.02 for the thermotherapy group, 23.06 ± 3.54 for the control group, and 26.03 ± 3.70 for the comparison group. ANCOVA indicated significant differences among groups (F = 10.367, *p* < 0.001). Post hoc analysis showed that Healthcare provider Satisfaction was significantly higher in the cryotherapy group than in the control group (*p* < 0.05).

### 3.3. Adverse Events

No adverse events or unintended effects were reported in any group during the study period.

## 4. Discussion

This randomized controlled trial evaluated the effects of the Enhanced Thermoelectric Element Tourniquet (E-TEET; E-TEE Tourniquet) on pain, stress, and satisfaction during intravenous catheter insertion in preoperative inpatients. When the E-TEET was applied, pain measured before needle insertion differed significantly among the four groups; however, no statistically significant differences in pain during needle insertion were observed among the cryotherapy, thermotherapy, control, and latex tourniquet groups. In contrast, stress levels during and after catheter insertion differed significantly among the groups, and both patient satisfaction and healthcare provider satisfaction also showed significant differences across all four groups.

The significant differences in pain observed immediately after tourniquet application and before venipuncture suggest that the E-TEET used in this study may have a favorable effect as a venous compression device. However, no significant differences were found in pain during or after venipuncture, when the needle penetrated the skin. Although pain during and after venipuncture was lower in the cryotherapy group than in the other groups, the absence of statistical significance indicates that the magnitude of the analgesic effect on needle insertion pain was limited. This finding is consistent with previous studies reporting inconsistent analgesic effects of thermal stimulation during short invasive procedures [24,26]. Given the brief duration and minimal tissue injury associated with intravenous catheterization, these results suggest that temperature-based stimulation alone may be insufficient to produce a clinically meaningful reduction in needle insertion pain.

In contrast, both stress levels and satisfaction outcomes demonstrated clear benefits associated with cryotherapy. Stress levels during and after venipuncture were significantly lower in the cryotherapy group compared with the control and comparison groups, and satisfaction was significantly higher. These findings indicate that cryotherapy may be effective in alleviating psychological tension before and after invasive procedures and in stabilizing autonomic nervous system responses. The significantly higher patient satisfaction observed in the cryotherapy group suggests that the intervention influenced procedural experience more through stress reduction and the resulting psychological comfort than through direct pain relief. In this study, participants in the cryotherapy group exhibited the lowest stress levels, and this emotional stability likely contributed to a more positive overall procedural experience. Previous studies have similarly reported that cryotherapy reduces stress and discomfort in hemodialysis patients, thereby improving patient satisfaction [12,24], supporting the present findings.

As a result, although the use of E-TEET during intravenous catheter insertion had limited effects on pain at the moment of needle insertion, it reduced pre-procedural pain and effectively decreased stress during and after the procedure. From a nursing practice perspective, stress reduction during procedures is clinically meaningful, as it can influence patient cooperation, procedural experience, and overall satisfaction, thereby representing an important contribution to nursing care.

Healthcare provider satisfaction was also highest in the cryotherapy group. However, this finding should be interpreted cautiously, as it may reflect healthcare providers’ subjective perceptions or expectancy effects based on recognizing patients’ reduced tension and emotional stabilization during the procedure, rather than an objective improvement in patient cooperation. Accordingly, healthcare provider satisfaction should be considered a supplementary outcome indicator. In the latex tourniquet group, although patients reported relatively higher pain scores, healthcare provider satisfaction remained at a moderate level. This may be explained by the practical advantages of latex tourniquets for nurses, such as ease of application and strong fixation, while simultaneously causing discomfort or constrictive sensations for patients due to increased skin pressure. These findings suggest that future tourniquet development should consider material selection and structural design improvements that balance nurse convenience with patient physical and emotional comfort.

Overall, although the E-TEET intervention did not result in statistically significant reductions in needle insertion pain, it demonstrated clear benefits in reducing stress and improving both patient and healthcare provider satisfaction, indicating its potential applicability in clinical nursing practice.

Several limitations should be acknowledged. The wide age range of participants and age differences among groups required covariate adjustment during analysis. In addition, the study was limited to intravenous catheter insertion, a minimally invasive procedure with a short duration, which may have constrained the observable effects of temperature-based interventions on pain and physiological responses. Furthermore, complete blinding was not feasible due to the nature of the intervention, which may have influenced subjective outcomes such as stress and satisfaction.

Despite these limitations, this study is meaningful in that it extends the role of the E-TEE Tourniquet beyond a simple hemostatic device to a non-pharmacological nursing intervention capable of integrating both cryotherapy and thermotherapy. In particular, the wireless power system enables rapid and convenient application by nurses during procedures, offering the potential to improve patients’ procedural experiences.

In conclusion, although the E-TEE Tourniquet did not produce significant differences in pain during intravenous catheter insertion, it demonstrated meaningful clinical applicability by reducing patient stress and improving patient and healthcare provider satisfaction. Future studies should establish standardized intervention parameters, including temperature and application duration, and further validate these findings across diverse clinical settings and patient populations.

## 5. Conclusions

The present study shows that the E-TEE Tourniquet had a limited effect on pain reduction during venipuncture, with no significant differences observed during or after needle insertion. However, cryotherapy significantly reduced stress levels and improved patient satisfaction during intravenous catheterization. These findings suggest that the E-TEE Tourniquet, particularly when used for cryotherapy, may serve as a useful non-pharmacological nursing intervention for alleviating psychological stress and enhancing patient experience in clinical practice.

## Figures and Tables

**Table 1:** Homogeneity of general characteristics across four groups (n = 128).

Characteristics	Category	E-TEET/C ^a^ (n = 31)	E-TEET/T ^b^ (n = 31)	Control ^c^ (n = 33)	Comparison ^d^ (n = 33)	χ^2^ or F	*p*
		Mean ± SD or n (%)	Mean ± SD or n (%)	Mean ± SD or n (%)	Mean ± SD or n (%)		
Gender	Male	10 (32.3)	10 (32.3)	17 (51.5)	14 (42.4)	3.456	0.327
	Female	21 (67.7)	21 (67.7)	16 (48.5)	19 (57.6)		
Age (y)		43.84 ± 13.31	40.77 ± 12.91	52.00 ± 12.72	53.55 ± 10.19	8.098	<0.001 (b < d) *
Height (cm)		163.71 ± 8.64	165.48 ± 8.78	162.39 ± 10.38	163.26 ± 9.37	0.617	0.606
Weight (kg)		67.45 ± 13.12	66.02 ± 12.95	64.94 ± 11.44	62.20 ± 14.61	0.926	0.430
Exercise (times/week)	<3	22 (71.0)	22 (71.0)	28 (84.8)	24 (72.7)	2.336	0.506
	≥3	9 (29.0)	9 (29.0)	5 (15.2)	9 (37.3)		
Smoking	Yes	4 (12.9)	8 (25.8)	6 (18.2)	7 (21.2)	5.766	0.450
	No	27 (87.1)	23 (74.2)	27 (81.8)	26 (78.8)		
IV insertion experience	≤1 week	7 (22.6)	10 (32.3)	9 (27.3)	13 (39.4)	8.328	0.501
	≤1 month	14 (45.2)	16 (51.6)	17 (51.3)	12 (36.4)		
	≤3 months	3 (9.7)	1 (3.2)	5 (15.2)	3 (9.1)		
	≤6 months	7 (22.6)	4 (12.9)	2 (6.1)	5 (15.2)		
Vein status	Excellent	3 (9.7)	4 (12.9)	1 (3.0)	3 (9.1)	10.491	0.573
	Good	6 (19.4)	5 (16.1)	5 (15.2)	2 (6.1)		
	Fair	14 (45.2)	15 (48.4)	11 (33.3)	13 (39.4)		
	Poor	6 (19.4)	5 (16.1)	10 (30.3)	9 (27.3)		
	Very poor	2 (6.5)	2 (6.5)	6 (18.2)	6 (18.2)		
Pain tolerance	Strong	4 (12.9)	8 (25.8)	7 (21.2)	8 (24.2)	7.276	0.608
	Moderate	17 (54.8)	17 (54.8)	13 (39.4)	14 (42.4)		
	Weak	6 (19.4)	6 (19.4)	9 (27.3)	7 (21.2)		
	Very weak	4 (12.9)	0	4 (12.1)	4 (12.1)		
Previous pain (NRS)		5.52 ± 1.79	5.48 ± 1.50	5.33 ± 2.01	5.15 ± 2.17	0.246	0.864
Previous Stress (NRS)		5.29 ± 2.18	4.87 ± 2.18	5.03 ± 2.01	5.18 ± 2.34	0.220	0.883

E-TEET/C ^a^: Enhanced Thermoelectric element Tourniquet–Cryotherapy group. E-TEET/T ^b^: Enhanced Thermoelectric element Tourniquet–Thermotherapy group. Control ^c^: Control Group (only E-TEE Tourniquet). Comparison ^d^: Comparison Group (Latex Tourniquet, LT). Previous pain: IV pain experience within 6 months. Previous Stress: IV stress experience within 6 months. * Bonferroni.

**Table 2:** Effects of Cryotherapy and Thermotherapy Using the E-TEET on Pain, Stress, Physiological Responses, and Satisfaction Across Four Groups (n = 128).

Variable	Category	E-TEET/C ^a^ (n = 31)	E-TEET/T ^b^ (n = 31)	Control ^c^ (n = 33)	Comparison ^d^ (n = 33)	F (*p*)
		Mean ± SD	Mean ± SD	Mean ± SD	Mean ± SD	
Pain	Pre-test (NRS)	0.42 ± 0.77	0.71 ± 0.86	0.55 ± 0.75	1.97 ± 1.13	21.148 ^†^ (<0.001) a, b, c < d *
	Insertion pain (NRS)	4.81 ± 2.84	4.87 ± 2.30	5.48 ± 1.92	5.58 ± 1.87	0.422 ^‡^ (0.737)
	Post test (NRS)	1.68 ± 1.25	2.58 ± 1.79	2.42 ± 1.33	3.15 ± 1.25	2.101 ^‡^ (0.104)
Pulse Rate (bpm)	Pre Pulse rate	75.06 ± 12.77	78.32 ± 10.79	75.82 ± 11.33	77.73 ± 13.29	1.335 (0.266)
	Insertion Pulse rate	76.48 ± 13.55	78.68 ± 10.28	77.55 ± 11.92	77.70 ± 18.09	0.659 (0.579)
	Post Pulse rate	76.29 ± 13.77	78.13 ± 9.89	77.21 ± 11.61	78.76 ± 13.13	1.261 (0.291)
SpO_2_	Pre SpO_2_	97.71 ± 1.32	98.06 ± 1.34	97.58 ± 1.42	97.30 ± 1.55	1.860 (0.140)
	Insertion SpO_2_	97.74 ± 1.50	97.90 ± 1.49	97.64 ± 1.50	97.06 ± 1.69	1.710 (0.168)
	Post SpO_2_	97.61 ± 1.41	98.03 ± 1.33	97.70 ± 1.59	97.30 ± 1.55	1.615 (0.189)
Stress	Pre-test (NRS)	2.90 ± 2.65	3.13 ± 2.78	3.73 ± 2.36	4.18 ± 2.43	1.143 (0.335)
	Insertion stress (NRS)	3.68 ± 2.57	4.68 ± 2.63	5.85 ± 2.45	5.48 ± 1.89	3.757 (0.013) a < c *
	Post test (NRS)	1.23 ± 1.12	2.06 ± 2.03	1.55 ± 1.44	2.73 ± 1.53	5.967 (<0.001) a < d *
Satisfaction	Patient	35.90 ± 7.08	31.68 ± 5.63	28.03 ± 5.74	23.21 ± 4.74	22.235 (<0.001) a > d *
	Healthcare provider	29.52 ± 5.97	26.39 ± 5.02	23.06 ± 3.54	26.03 ± 3.70	10.367 (<0.001) a > c *

E-TEET/C ^a^: Enhanced Thermoelectric element Tourniquet–Cryotherapy group. E-TEET/T ^b^: Enhanced Thermoelectric element Tourniquet–Thermotherapy group. Control ^c^: Control Group (only E-TEE Tourniquet). Comparison ^d^: Comparison Group (Latex Tourniquet, LT). NRS: Numeral rating scale. Pre pain: Pre-needle pain after tourniquet Insertion pain: Needle insertion pain. Post pain: Post-needle insertion pain. Pre pulse rate: Pre-needle pulse rate after tourniquet. Insertion pulse rate: Needle insertion pulse rate. Post pulse rate: Post-needle insertion pulse rate. bpm: beats per min. SpO_2_: Saturation of Percutaneous Oxygen. Pre SpO_2_: Pre-needle SpO_2_ after tourniquet Insertion. SpO_2_: Needle insertion SpO_2_. Post SpO_2_: Post-needle insertion SpO_2_. Pre Stress: Pre-needle stress after tourniquet Insertion. Stress: Needle insertion stress. Post Stress: Post-needle insertion stress. ^†^: ANCOVA (covariate values: age = 47.70) ^‡^: ANCOVA (covariate values: age = 47.70, pre pain = 0.92). * Bonferroni.

## Data Availability

The data presented in this study are available on request from the corresponding author due to ethical and privacy considerations related to participant confidentiality.

## Supplementary Materials

The following supporting information can be downloaded at: https://www.mdpi.com/article/10.3390/nursrep16010017/s1, Supplementary Material S1: Enhanced Thermoelectric Element Tourniquet(E-TEET); Supplementary Material S2: Intervention process.

## Public Involvement Statement

Patients were involved as study participants in this randomized controlled trial; however, they were not involved in the design, conduct, analysis, or reporting of the research.

## Guidelines and Standards Statement

This manuscript was drafted against the Consolidated Standards of Reporting Trials (CONSORT) 2010 Statement for randomized controlled trial research. The CONSORT checklist and flow diagram are provided as Supplementary Materials.
