# Supplementary material for: Effects of Cryotherapy and Thermotherapy Using an E-TEET on Pain, Stress, and Satisfaction Among Patients and Healthcare Providers During Intravenous Catheterization: A Randomized Controlled Trial
Source: Nurs Rep. 2026 Jan 7;16(1):17. doi: 10.3390/nursrep16010017 (PMC12844899; doi:10.3390/nursrep16010017)

Supplement 1-a. Enhanced Thermoelectric Element Tourniquet(E-TEET)

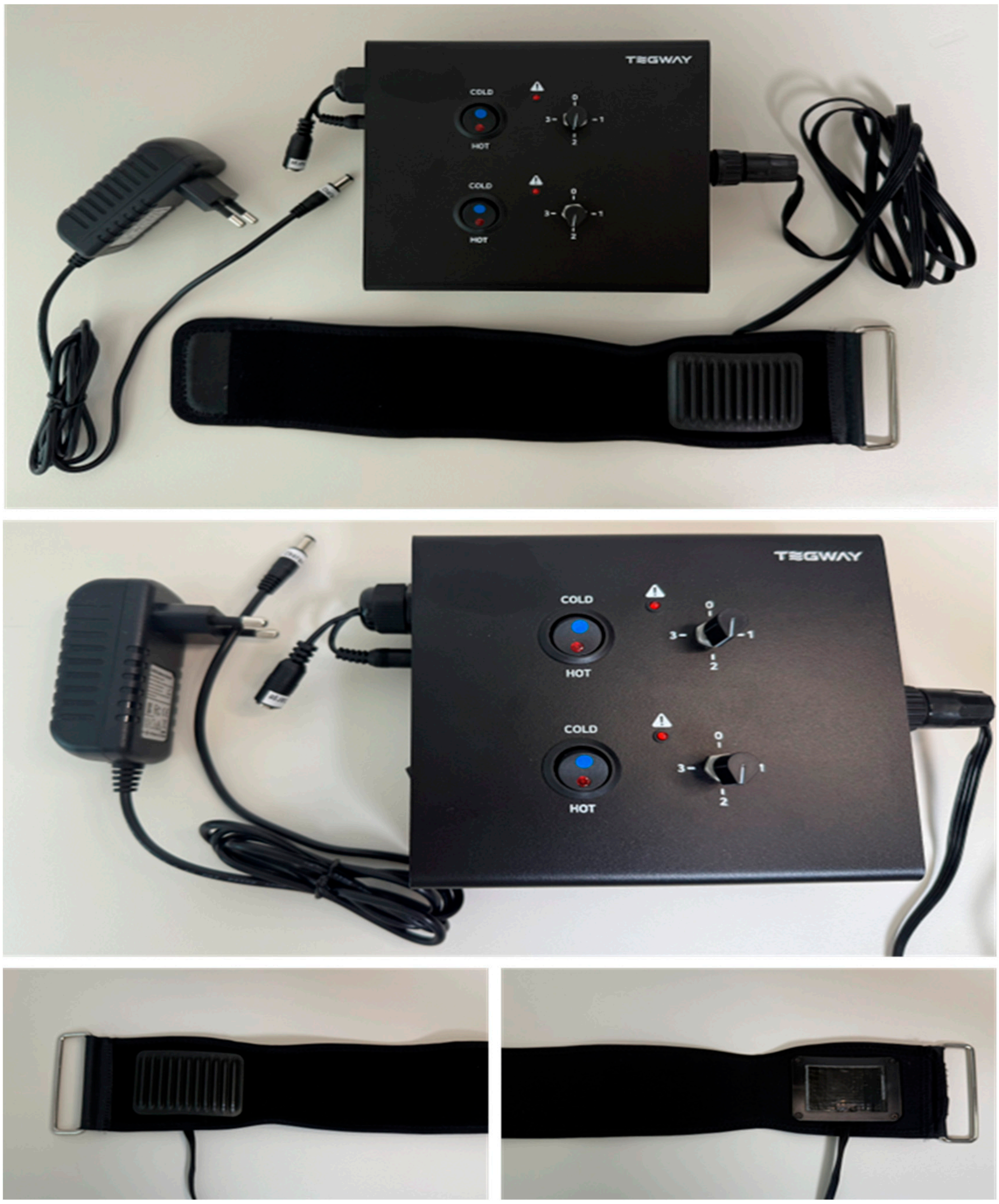

Supplement1-b. Applying E-TEE Tourniquet intervention during intravenous catheterization

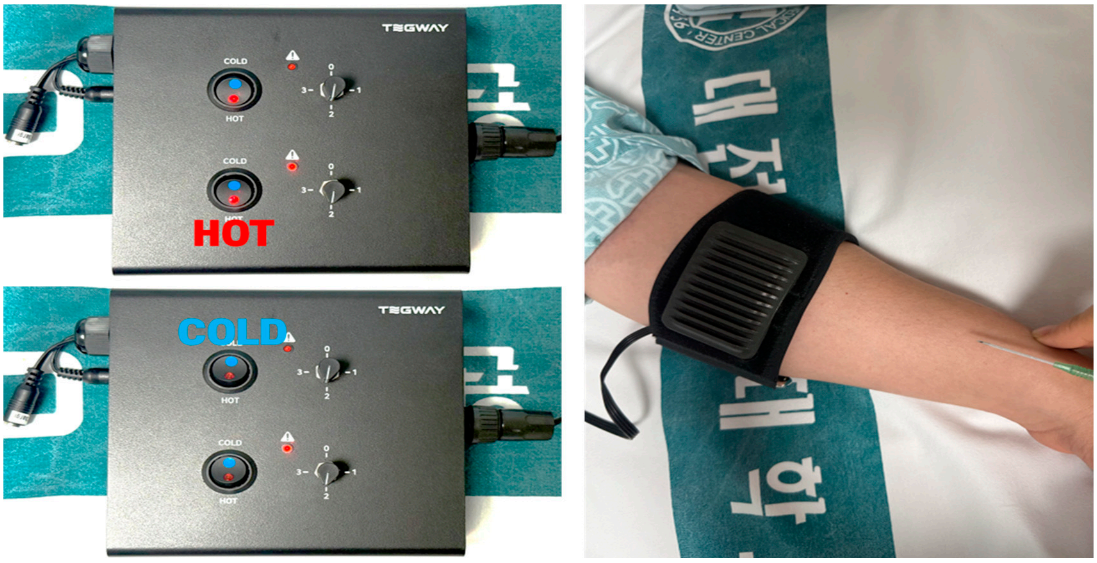

Supplement1-c. Applying Latex Tourniquet during intravenous catheterization

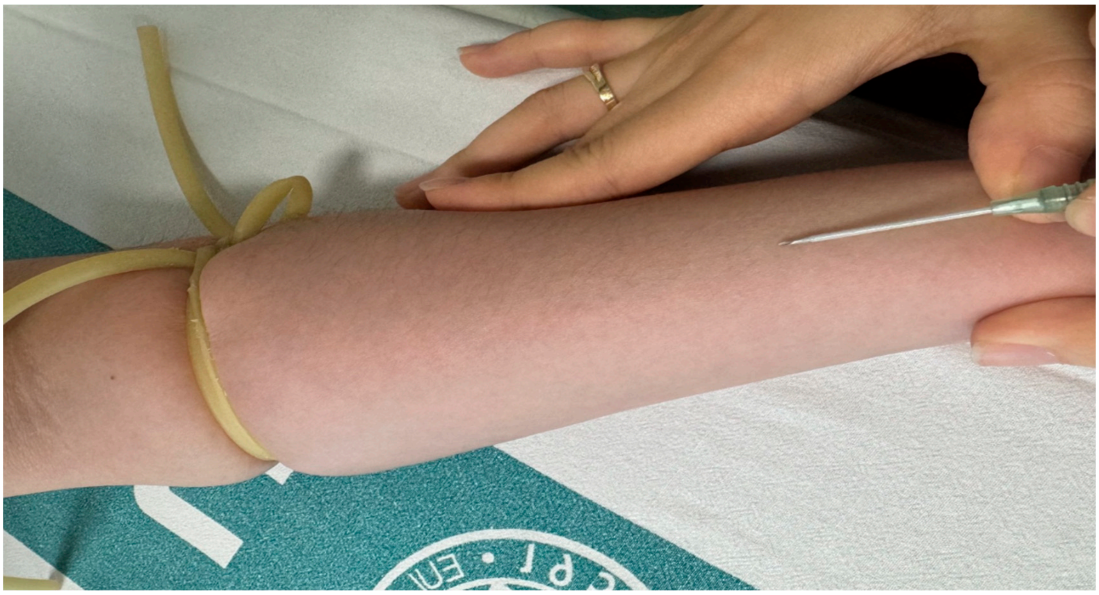

Supplement: Supplementary file 1 [file nursrep-16-00017-s001.zip › supplement 1(fianl).pdf]
