# Supplementary material for: Effects of Cryotherapy and Thermotherapy Using an E-TEET on Pain, Stress, and Satisfaction Among Patients and Healthcare Providers During Intravenous Catheterization: A Randomized Controlled Trial
Source: Nurs Rep. 2026 Jan 7;16(1):17. doi: 10.3390/nursrep16010017 (PMC12844899; doi:10.3390/nursrep16010017)
Supplement: Supplementary file 1 [file nursrep-16-00017-s001.zip › supplement 2(fianl).pdf]

supplement 2. Intervention Process

No	Procedure	Operator	Writer	Measurement items
**1**	The research assistant explained the research procedures using a written information sheet and obtained written consent from the participants.	Research Assistant	Participants	
**2**	The research assistant had the participants complete a questionnaire collecting demographic information and past experiences of pain and stress (NRS) during intravenous catheterization.	Research Assistant	Participants	·Pain (NRS)<br>·Stress (NRS)
**3**	The research assistant applied a tourniquet 10-12 cm above the planned catheterization site and placed a pulse oximeter on the opposite arm.	Research Assistant		
**4**	Following tourniquet application, participants were instructed to report their perceived levels of pain (pre-pain) and stress (pre-stress) prior to the intravenous injection. Pulse oximeter readings were recorded by the researcher before the injection.	Researcher	Researcher<br>Participants	·Pain(NRS)<br>·Stress(NRS)<br>·Pulse rate(bpm)<br>·SpO_2
**5**	Sealed envelopes containing randomization results for the four groups were opened sequentially according to the participant's registration order to determine group assignment.	Researcher		
**6**	The research assistant activated the temperature setting on the Enhanced Thermoelectric Element Tourniquet(E-TEET) according to the participant's assigned group as follows:<br>• **E-TEET-warm group:** The HOT button was activated at level 2 (40–45°C) for a minimum of 10 seconds and up to a maximum of 30 seconds, depending on the time required for successful catheterization.<br>• **E-TEET-cold group:** The COLD button was activated at level 2 (0–10°C) for a minimum of 10 seconds and up to a maximum of 30 seconds, depending on the time required for successful catheterization.<br>• **E-TEET-band group:** No temperature setting was activated after the tourniquet was applied.<br>• **Control group:** A standard elastic tourniquet was used without any temperature modulation.	Researcher		
**7**	The research assistant monitored the participant for any temperature-related discomfort for approximately 5 seconds. If no discomfort was reported, the catheterization site was disinfected with an alcohol swab, and catheterization was performed using an 18-gauge intravenous catheter. Upon successful catheterization, the tourniquet was removed, a saline flush was administered to confirm catheter patency, and the site was secured with fixation tape.	Research Assistant		
**8**	The researcher continuously monitored and recorded the participant's pulse rate and oxygen saturation levels at the time of needle insertion for intravenous catheterization, as well as upon completion of the procedure.	Research Assistant	Researcher	·Pulse rate (bpm)<br>·SpO_2
**9**	After completing all procedures, the participants completed a questionnaire assessing their perceived pain (insertion pain) and stress (insertion stress) at the time of needle insertion, perceived pain (post-pain) and stress (post-stress) following intravenous catheterization, and satisfaction with the tourniquet application.	Research Assistant	Participants	·Pain (NRS)<br>·Stress (NRS)<br>·Tourniquet Satisfaction (NRS)<br>Participant satisfaction
**10**	Finally, the research assistant completed a questionnaire assessing practitioner's satisfaction with the tourniquet experience during the catheterization procedure.	Research Assistant	Research Assistant	·Tourniquet Satisfaction (NRS) practitioner's satisfaction
